# Can internship programs affect nursing students’ critical thinking disposition, caring behaviors, and professional commitment?

**DOI:** 10.1186/s12912-024-02089-3

**Published:** 2024-06-21

**Authors:** Zahra Sarkoohi, Monirsadat Nematollahi, Mahlagha Dehghan, Roghayeh Mehdipour-Rabori, Zohreh Khoshnood, Peiman Parandeh-Afshar, Jamileh Farokhzadian

**Affiliations:** 1 Reproductive and Family Health Research Center, Kerman University of Medical Sciences, Kerman, Iran; 2 Nursing Research Center, Kerman University of Medical Sciences, Kerman, Iran; 3 Health in Disasters and Emergencies Research Center, Institute for Futures Studies in Health, Kerman University of Medical Sciences, Kerman, Iran

**Keywords:** Critical thinking disposition, Caring behaviors, Internship programs, Professional commitment, Nursing students

## Abstract

**Background:**

Nursing students are given opportunities to develop critical thinking disposition, caring behaviors, and professional commitment through clinical training. Therefore, nurse educators should move away from traditional methods toward new ones, such as internship programs in clinical training. This study assessed the effect of nursing internship programs on senior undergraduate nursing students’ critical thinking disposition, caring behaviors, and professional commitment.

**Methods:**

This quasi-experimental study was conducted using a pretest-posttest design but with no control group. The study sample included 46 senior students enrolled in nursing internship programs. A demographic questionnaire, the Critical Thinking Disposition Inventory (CTDI), the Caring Assessment Report Evaluation (Care-Q), and the Nursing Professional Commitment Scale (NPCS) were used to collect data before and five months after the nursing internship programs were implemented.

**Results:**

The study findings revealed that the senior nursing students’ caring behaviors improved, but the total scores of critical thinking disposition and professional commitment did not change significantly after the nursing internship programs (*p* > 0.05).

**Conclusion:**

According to the results, nurse educators are recommended to adopt strategies to improve the effectiveness of internship programs on critical thinking disposition and professional commitment among senior undergraduate nursing students.

## Introduction

One of the most important aspects of practical training is exposing nursing students to a clinical learning environment. In this way, nursing students receive clinical training in a complex clinical environment that allows them to convert theoretical knowledge into a variety of mental, psychological, and psychomotor skills [1]. They encounter various problematic situations related to treatment, disease symptoms, and patient care in clinical settings, with varying levels of complexity, importance, and urgency. Therefore, in these situations, creative, analytic, and critical thinking is an essential nursing skill for nursing practice [2].

Critical thinking is the purposeful, self-regulatory judgment that leads to interpretation, analysis, evaluation, and inference, as well as the explanation of the evidential, conceptual, methodological, and contextual considerations that are used to make judgments [3, 4]. Critical thinking requires a set of skills and attitudes or dispositions. Critical thinking is reflective and rational thinking that significantly impacts decision-making regarding our beliefs and actions [3]. Skills are the cognitive component (such as analysis, interpretation, inference, explanation, evaluation, and self-regulation) and attitudes are the affective component (such as open-mindedness, inquisitiveness, truth-seeking, and being analytical, systematic, and self-confident in reasoning). It is necessary to place greater emphasis on disposition when teaching thinking because disposition is responsible for the efficiency of such teaching. In particular, it is influential on the use of the skill taught; that is, on the extent to which those thinking skills are utilized. Dispositions might be required not only for executing such thinking but also for learning it. An important set of prior dispositions should be considered before starting instruction which may lead to improved learning of skills and enhanced critical thinking [5]. Experts have placed a greater emphasis on critical thinking skills, with the attitudinal aspect of critical thinking receiving less attention [6].

In addition, nursing students must develop professional commitment while studying at a university. Since nursing is a practice-based profession, clinical learning environments play an important role in improving professional abilities and preparing nursing students to enter the nursing profession as competent and committed nurses [1]. Professional commitment is the sense of identity or dependence on a specific profession, the way a person thinks about his or her profession, and the motivation to work in that profession; people with higher levels of commitment strive to promote professional values and are more responsible [7]. Professional commitment is defined as “a belief in and acceptance of the values of the chosen profession, effort to actualize these values, desire to improve himself/herself and determination to remain a member of that profession” [8]. Professional commitment connects individuals to the professions they choose and believe in, their attempts to reach values, the desire to develop themselves, and the determination to pursue their membership [9]. Sources of stress and conflict can be identified when the students find it difficult to adapt to their own situations within the profession, or even when they are not satisfied with their career choice [8].

Nursing students are expected to have a variety of skills, including caring behaviors. Caring behaviors are considered important in nursing education, especially as students begin to learn about core nursing values and the nature of the profession. It is also expected that students in their final years would place higher value on caring behaviors than first-year students [10]. Caring behaviors may take many forms, including words, thoughts, feelings, looks, actions, movements, gestures, body language, touch, acts, procedures, and/or information [11].

Researchers reported that nursing internship programs are effective for objective educational nursing programs, and they can create a learning environment for nursing students to develop critical thinking, professional commitment, caring behaviors, and professionalism [8, 12]. Nursing internship programs and the use of mentors in nursing education were developed in the late 1970s to respond to an acute shortage of nurses and to bridge the gap between educational and practical settings. The programs provide senior nursing students with a paid internship in clinical practice settings, where they work under the supervision of nurse instructors. The programs aim to improve the interns’ perception of their readiness for professional nursing practice [13]. Since 1992, the programs have been regarded as an innovation in the nursing education structure to improve the quality of nursing education and provide clinical services in nursing [14]. However, they have not been implemented successfully in Iran. The programs have recently been added to the nursing curriculum in Iran and piloted in a small number of universities in 2018. Students participate in a two-semester nursing internship program in the senior year [8].

The literature review showed that new nursing graduates who attended programs with no internship opportunities did not acquire the necessary capacity to function efficiently in clinical settings, lacked competence when undertaking clinical practice, demonstrated stress related to competence and confidence, made mistakes, and adjusted to a new workplace. Furthermore, these graduates struggled with task organization and completion as well as the quality of work [15]. Another study reported that reflective training during the internship period improved nursing students’ critical thinking disposition and promoted their readiness for clinical practices in the rapidly changing healthcare environment [16]. A study in Turkey showed that internship programs significantly increased the professional commitment and academic burnout of nursing students [8]. Other studies have shown that nursing internships have the potential to increase self-confidence, enhance clinical knowledge and skills, reduce stress and anxiety [17], help retain the job, and reduce the gap between theory and practice [13].

Given the importance of variables such as professional commitment, critical thinking disposition, and caring behaviors as well as the scarcity of related studies on the effectiveness of internship programs in the context of Iran, this study assessed the effect of nursing internship programs on professional commitment, critical thinking disposition, and caring behaviors of senior nursing students.

## Methods

### Study design

This quasi-experimental study used a pretest-posttest design with no control group and was conducted on one group of senior nursing students.

### Sample and setting

The study took place in a large hospital and a nursing school affiliated with Kerman University of Medical Sciences in the Southeast of Iran. According to Iranian academic law, a bachelor’s degree includes four years of study and is based on a semester system. As a result, students must pass theoretical and clinical courses at universities and educational hospitals in each academic year. The inclusion criterion was senior nursing students who passed the Objective Structured Clinical Examination (OSCE) regarding nursing skills. The exclusion criteria were failure to complete the questionnaires for any reason and not participating in one or more internship courses. The census method was used to select eligible students. Power analysis calculations with G*Power software indicate that (power = 90%, *p* = 0.05, number of groups = 1, and number of measurements = 3) 55 participants would be needed to detect an effect size of 0.2. Totally, 60 samples were assessed for eligibility, of which, 57 eligible participants finished the study.

### Instruments

Four instruments were used to collect data. First, a sociodemographic characteristics form was used to collect information about age, academic grade point average (GPA), gender, marital status, work experience, and history of employment.

The second tool was the Critical Thinking Disposition Inventory (CTDI) designed by Facione based on the critical thinking scale (1990). CTDI contains 33 items and three subscales, including innovation or creativity, cognitive maturity or perfection, and mental engagement or commitment. The CTDI is scored on a 5-point Likert scale including strongly disagree = 1, disagree = 2, no idea = 3, agree = 4, and strongly agree = 5. The total score of critical thinking disposition is obtained by summing the scores of the three subscales. A score equal to or higher than 135.31 shows strong critical thinking disposition, from 108.91 to 135.30 indicates moderate critical thinking disposition, and a score equal to or lower than 108.90 shows poor critical thinking disposition [18]. Ricketts (2003) confirmed the reliability of CTDI by reporting the Cronbach’s alpha coefficient of 0.79 for creativity, 0.89 for commitment, 0.75 for maturity, and 0.86 for the whole inventory [19]. In Iran, the reliability of CTDI was 0.71 using internal consistency [20].

The third tool used to collect data was the Nursing Professional Commitment Scale (NPCS) initially designed by Lachman and Aryana (1968) [21]. NPCS includes 26 items regarding the domains of nursing perception (6 items, items 1 to 6), nursing job satisfaction (4 items, items 7 to 10), engagement with the nursing profession (6 items, items 11 to 16), and self-sacrifice for the nursing profession (6 items, items 17 to 26). The NPCS is rated on a 5-point Likert scale including strongly disagree = 1, disagree = 2, no idea = 3, agree = 4, and strongly agree = 5. The scores range from 26 to 130, where scores of 26–43, 44–86, and 87–130 indicate low, moderate, and high professional commitment, respectively. The NPCS designers used internal consistency to determine the reliability of the scale and reported a Cronbach’s alpha of 0.86 for the whole scale. In Iran, Shali et al. confirmed the validity of NPCS using qualitative content validity and a survey from 12 experts and determined its reliability by internal consistency (α = 0.74) [22].

The fourth tool was the Caring Assessment Report Evaluation (Care-Q) developed by Larson (1981) as a self-assessment tool. The Care-Q is the most frequently used instrument for assessing caring behaviors in the world and hence the most appropriate instrument for international comparison [23]. The Persian version of the Care-Q include 57 items and six sub-skills: being accessible (6 items), explaining and facilitating (9 items), comforting (11 items), anticipating (5 items), trusting relationships (18 items), monitoring and following-up (8 items). The items are rated on a five-point Likert scale ranging from least important (1) to most important (5). The minimum score on this scale is 57, while the maximum attainable score is 285. The Persian version of this questionnaire was validated by Pashaee et al. (2014) using the back translation method. The experts’ viewpoints were used to determine the content validity, and the test-retest method was applied to determine the reliability (*r* = 0.87) [24].

### Nursing internship programs and data collection procedure

The study was conducted from September 2019 to January 2020. Data were collected using anonymous, self-report, and structured questionnaires. To collect data, one of the researchers went to the study setting and distributed the questionnaires among students in the pretest (before the internship) and posttest (five months after the internship) phases. Before starting the internship course, students were informed about the objective and the procedure of the course, and its contribution to professional knowledge and skills.

To design the internship programs, several meetings were held with the dean and vice-chancellor of the faculty and the heads of the departments at the nursing school. They discussed the development of a comprehensive program and its various components. The internship programs were then designed with the participation of faculty members as follows:

1. In the skill lab, nursing principles and skills were taught to the students.
2. All students were required to pass the OSCE before being accepted into the internship programs.
3. According to the nursing credits in the senior year, the Afzalipour Hospital was selected to implement the internship programs.
4. Students were divided into groups of 3–4 members in the target wards with coordination of the head nurses in different shifts (morning, evening, and night).
5. Several meetings were held with the head of the hospital, nurse managers, supervisors, and head nurses of the target wards to coordinate the implementation of the designed programs.
6. As an instructor, the head nurse of each ward supervised the students’ theoretical and practical education. They are employed by Kerman University of Medical Sciences.
7. The school of nursing appointed an instructor (a nursing professor) from each department to supervise clinical education and resolve the educational challenges of the students.
8. The daily lesson plan and practical and instructional tasks were provided to the students.
9. After the programs were completed, the OSCE was conducted for each course according to the educational content of that course. This was the same OSCE as before the internship.
10. At the beginning, the students, head nurses, and the clinical supervisor of the faculty were given the checklist for the OSCE of each training programs, and they were all made aware of the training and evaluation process.

### Statistical analysis

Data were analyzed using SPSS software V_20_, descriptive statistics (frequency, percentage, mean, and standard deviation), and inferential statistics (paired-t test, Wilcoxon test, and McNemar test). The significance level was set at ≤ 0.05.

## Results

### Demographic information

Of 57 eligible students, 11 nursing students did not complete the questionnaires in the post-test; therefore, 46 students completed the study (response rate = 80.70). There were no significant differences regarding demographic data between those who completed the post-test and those who did not (*P* > 0.05). The results showed that the mean age of participants was 23.55 ± 4.9 years (Min = 21 and Max = 45). The mean score of the participants’ academic grade point average (GPA) was 16.07 ± 1.08 (Min = 14 and Max = 18.51). About 58.7% (*n* = 27) of the participants were female and 69.6% (*n* = 32) were married. Besides, 39.1% (*n* = 18) of the participants had work experience as a nursing student, and 10.9% (*n* = 5) of the participants had work experience as a practical nurse.

As it is presented in Table 1, critical thinking disposition and its domains improved after the nursing internship course. Moreover, 13% (*n* = 6) of the study participants had a weak critical thinking disposition, 74% (*n* = 34) had a moderate critical thinking disposition, and 13% (*n* = 6) had a high critical thinking disposition before the intervention while it was moderate in 89.1% (*n* = 41) and high in 10.9% (*n* = 5) of the participants after the intervention (Wilcoxon test = -1.11, *p* = 0.27). Subgroup analysis showed there were no significant differences regarding critical thinking disposition and its domains between those who had work experience as a student in the hospital and those who did not, before and after the intervention (*P* > 0.05). In addition, there were no significant differences regarding critical thinking disposition and its domains between those who had work experience as a practical nurse and those who did not, before and after the intervention (*P* > 0.05).

**Table 1:** Comparison of the mean scores of critical thinking disposition and its dimensions before and after the nursing internship

Time	Before intervention	After intervention		
Variable	M ± SD	M ± SD	Statistical test	***P***-value
Innovation or creativity	42.09 ± 4.36	43.0 ± 2.95	Z = -0.66	0.51
Cognitive maturity or perfection	29.35 ± 3.84	29.87 ± 2.69	t = 0.82	0.42
Mental engagement or commitment	49.87 ± 5.47	52.28 ± 4.44	Z = -2.15	0.03
Total critical thinking disposition	121.30 ± 11.16	125.15 ± 7.99	Z = -1.45	0.15

Z = Wilcoxon test, t = paired t test

As Table 2 represents, the participants’ professional commitment and its domains did not improve significantly after the nursing internship course (*p* > 0.05). In addition, professional commitment was moderate in 32.6% (*n* = 15) and high in 67.4% (*n* = 31) of the study participants before the intervention while it was moderate in 21.7% (*n* = 10) and high in 78.3% (*n* = 36) of the participants after the intervention (McNemar test p-value = 0.23). Subgroup analysis showed there were no significant differences regarding professional commitment and its domains between those who had work experience as a student in the hospital and those who did not, before and after the intervention (*P* > 0.05). In addition, there were no significant differences regarding professional commitment and its domains between those who had work experience as a practical nurse and those who did not, before and after the intervention (*P* > 0.05).

**Table 2:** Comparison of the mean scores of professional commitment and its dimensions before and after the nursing internship

Time Variable	Before intervention M ± SD	After intervention M ± SD	Statistical test	*P*- value
Nursing perception	18.11 ± 3.92	18.78 ± 4.82	Z = -1.10	0.27
Nursing job satisfaction	14.04 ± 3.33	14.20 ± 4.35	Z = -0.87	0.38
Engagement with the nursing profession	23.09 ± 3.56	24.35 ± 2.65	Z = -1.25	0.21
Self-sacrifice for the nursing profession	33.04 ± 5.62	34.70 ± 4.96	Z = -0.34	0.74
Total professional commitment	88.28 ± 13.69	92.02 ± 14.50	t = 1.51	0.14

Z = Wilcoxon test, t = paired t test

As shown in Table 3, the participants’ caring behavior and all its domains improved significantly after the nursing internship course (*p* > 0.05). Subgroup analysis showed there were no significant differences regarding caring behavior and its domains between those who had work experience as a student in the hospital and those who did not, before and after the intervention (*P* > 0.05). In addition, there were no significant differences regarding caring behavior and its domains between those who had work experience as a practical nurse and those who did not, before and after the intervention (*P* > 0.05).

**Table 3:** Comparison of the mean scores of caring behavior and its dimensions before and after the nursing internship

Time	Before intervention	After intervention		
Variable	M ± SD	M ± SD	Wilcoxon test	***P***-value
Being accessible	24.80 ± 3.30	26.65 ± 2.44	-3.15	0.002
Explaining and facilitating	36.30 ± 4.49	39.65 ± 5.25	-3.57	< 0.001
Comforting	42.83 ± 7.32	47.28 ± 7.15	-3.71	< 0.001
Anticipating	18.59 ± 3.44	20.98 ± 3.25	-3.44	0.001
Trusting relationships	68.20 ± 10.94	74.63 ± 9.73	-3.48	0.001
Monitoring and following-up	31.28 ± 5.78	35.15 ± 4.93	-4.0	< 0.001
Total caring behavior	222.0 ± 31.05	244.35 ± 30.0	-4.48	< 0.001

## Discussion

The findings from this study showed there were no significant changes in professional commitment and critical thinking disposition scores among senior undergraduate nursing students following the internship programs. Moreover, the results indicate the positive impact of the internship program on caring behaviors.

Regarding critical thinking disposition, the results indicated that the total score of critical thinking disposition and its domains increased after the nursing internship. Zhang et al. (2017) showed that students’ critical thinking disposition improved during internship programs and students might become more organized, orderly, focused, and diligent in their clinical practices [16]. Wu et al. demonstrated critical thinking disposition had a positive effect on self-decision, self-cognition, self-confidence, and self-responsibility. Thus, they emphasized the importance of improving nursing students’ critical thinking ability [25]. Researchers in a systematic review reported that critical thinking could be achieved in experimental teaching conditions that have been significantly related to positive impacts on patient outcomes, quality of care, and nursing knowledge advancements [26].

The results of the present study showed that the nursing internship programs significantly improved caring behaviors among nursing students. Accordingly, thoughts, feelings, looks, actions, movements, gestures, body language, touch, acts, procedures, and information are among the features and actions of caring behaviors that seem to be the central core of the nursing practice required to be reinforced during the nursing educational programs [27]. The emphasis of educators and clinical nurses on learning and prioritizing caring behaviors in clinical education is probably the reason for the greater impact of the internship programs on caring behaviors. On the other hand, students try to learn better caregiving behaviors in clinical education to enter different public and private settings faster. Besides, caring behaviors are directly related to nursing practice and nurses’ daily clinical work. Thus, nurses, nurse educators, and people have high expectations from nursing students when it comes to care. In contrast to the findings of the present study, an Iranian study found that internship programs were ineffective in improving students’ technical and communication skills and students were dissatisfied with them. The mean score for technical and communication skills was significantly higher in the control than in the intervention group. According to the researchers, the presence of an instructor following a traditional method and the provision of necessary training to students during the internship programs may be the reasons for the higher score in the control group. The students in the intervention group, on the other hand, had less access to the instructors and had to rely on the department’s staff to learn the skills. As the role of mentorship for these clinical nurses was not defined and they were not responsible for student’s education, they made no effort to train students. Nursing staff also required students to perform tedious and repetitive tasks such as vital sign control and sampling, among other things, which could lead to student dissatisfaction [14].

The findings of the current study also revealed that the internship programs did not affect the professional commitment of the participants. The results of a study reported that these programs increased the commitment of senior nursing professionals. Since the internship programs have aimed to prepare students for the profession and provide them with core competencies of nursing, they will affect future job quality [8]. The results of the present study imply that nursing students could not perceive themselves to be more competent and motivated after the internship course. In a study, it was emphasized that professional commitment is the main source of positive professional behavior and professional competence and the presence of nurses with a higher professional commitment improves the public image of the nursing profession for patients and society. Therefore, strategies are required to guide nurses toward achieving the professional objectives of nursing and provide quality nursing services [28]. The present study suggests that one of the nurse educators’ major responsibilities is to train nursing students properly in clinical settings, resulting in higher enthusiasm and motivation for learning the components of professional commitment.

In general, the design and implementation of these programs did not affect nursing students’ professional commitment and critical thinking. Accordingly, inadequate access to educational and welfare facilities, lack of cooperation among the healthcare team, presence of students in various clinical wards, shortage of motivated instructors, lack of appropriate educational fields, lack of institutionalized evaluation systems, disregard of clinical training, and entrusting students to inexperienced nurses in the wards are all issues that need to be addressed. Therefore, since the correct implementation of the internship programs can have valuable results, changes should be made in the way they are implemented. The factors mentioned above should be studied in terms of psychometrics, method, evaluation, and implementation to adopt a proper policy so that students with basic competencies are prepared for community-based and clinical roles. Educational administrators and policymakers are expected to develop and implement these programs, taking into account the weaknesses and strengths of the programs as well as the perspectives of students and educators.

### Limitations

There were two limitations in this study. Firstly, as the number of participants recruited in this study was limited, we could not have any control groups to compare the results with the intervention group. Secondly, nursing internship programs were conducted only in a few nursing education programs in Iran, and limited literature was found to compare the results with.

## Conclusion

This study revealed that the caring behaviors of senior nursing students improved after participating in the nursing internship programs. However, the total scores of critical thinking disposition and professional commitment of students did not change significantly after these programs. For this reason, senior students may require modified nursing internship programs or different approaches to enhance critical thinking disposition and professional commitment compared with those tested in this study. Educational programs focusing on improving critical thinking and professional nursing competence can play a fundamental role in enhancing the abilities and commitment levels of nursing students [5]. Therefore, appropriate approaches in internship programs and educational curricula may be essential for fostering greater critical thinking skills and professional commitment among senior nursing students. Moreover, further studies using mixed methods and longitudinal designs are required to explore nursing students’ perceptions of the effectiveness of nursing internship programs in diverse cultures and contexts. Studies evaluating innovations in curriculum and teaching methods seem to improve clinical learning. Furthermore, exploring diversity in assessment processes and the challenges faced by students and trainers in developing and evaluating case studies can provide valuable insights for improving educational methods in nursing programs. Integrating global learning experiences in nursing education can also contribute to the development of cultural competence among students, preparing them for effective work in multicultural healthcare environments.

## Data Availability

The data are available upon request to the corresponding author after signing appropriate documents in line with ethical application and the decision of the Ethics Committee.

## Abbreviations

OSCE: Objective Structured Clinical Examination
GPA: grade point average
